# Exploring MAP2K3 as a prognostic biomarker and potential immunotherapy target in glioma treatment

**DOI:** 10.3389/fneur.2024.1387743

**Published:** 2024-06-13

**Authors:** Bei Pu, Shi Feng, Lijuan Gu, Daniel Smerin, Zhihong Jian, Xiaoxing Xiong, Liang Wei

**Affiliations:** ^1^Department of Neurosurgery, Renmin Hospital of Wuhan University, Wuhan, China; ^2^Transplantation Health Management Center, Sichuan Taikang Hospital, Chengdu, China; ^3^Central Laboratory, Renmin Hospital of Wuhan University, Wuhan, China; ^4^Department of Neurosurgery, University of Central Florida College of Medicine, Orlando, FL, United States

**Keywords:** glioma, immunotherapy, MAP2K3, tumor immune microenvironment, prognosis

## Abstract

Glioma, the most prevalent primary brain tumor in adults, is characterized by significant invasiveness and resistance. Current glioma treatments include surgery, radiation, chemotherapy, and targeted therapy, but these methods often fail to eliminate the tumor completely, leading to recurrence and poor prognosis. Immune checkpoint inhibitors, a class of commonly used immunotherapeutic drugs, have demonstrated excellent efficacy in treating various solid malignancies. Recent research has indicated that unconventional levels of expression of the MAP2K3 gene closely correlates with glioma malignancy, hinting it could be a potential immunotherapy target. Our study unveiled substantial involvement of MAP2K3 in gliomas, indicating the potential of the enzyme to serve as a prognostic biomarker related to immunity. Through the regulation of the infiltration of immune cells, MAP2K3 can affect the prognosis of patients with glioma. These discoveries establish a theoretical foundation for exploring the biological mechanisms underlying MAP2K3 and its potential applications in glioma treatment.

## Background

Glioma is the most common primary brain tumor and is a general term for a large group of intracranial primary tumors that occur from glial cells derived from neural ectoderm. Gliomas can be categorized as grade I–IV according to the World Health Organization (WHO) grading standards. Glioblastoma (GBM) is a high-grade glioma, defined as WHO grade IV, and is one of the most lethal gliomas. It makes up 70–75% of all diffuse gliomas, and GBM patients have a median survival time of about 14–17 months. The main treatment options are surgery, radiotherapy, temozolomide (TMZ) chemotherapy and radiotherapy combined with TMZ chemotherapy, but none of the treatment outcomes are satisfactory (1).

The surgical elimination of the tumor, followed by chemotherapy and radiation therapy, is the conventional treatment for glioblastoma. As a result of the extremely aggressive characteristics of glioma cells, complete removal of the tumor is currently difficult to achieve (2). The potential mechanisms of glioma migratory invasion remain to be further investigated (3). Immunotherapy is able to inhibit tumor growth and spread by activating the patient’s own immune system. Common immune cell types in glioma patients include T cells, B cells, dendritic cells, and natural killer cells; which are closely related to tumor immune escape and drug resistance (4). Immunotherapy has been demonstrated to improve patient survival as well as quality of life in glioma patients (5). Additionally, immunotherapy may be used together with more established treatments like radiation and chemotherapy to maximize therapeutic outcomes. Despite the clinical effectiveness of immunotherapy in the management of gliomas, additional investigation is required to address potential issues with immune escape and drug resistance (5).

Mitogen-activated protein kinase 3 (MAP2K3) is a member of the bispecific protein kinase kinases (MKK) group, which is found in the Mitogen-activated protein kinase pathway (MAPK) (6). The MAP2K3 protein was first discovered in 1996, and current research has focused on its role as an activator of the p38-MAPK signaling pathway (7). In the therapeutic studies of esophageal squamous cell carcinoma, MAP2K3 inhibitors have been reported to be effective in inhibiting cell growth. MAP2K3 can mediate cellular responses to external stimuli by phosphorylating and activating the p38-MAPK signaling pathway (8). When cells are subjected to external stimuli, activated MAP3K activates MAP2K3 to phosphorylate and activate the p38-MAPK signaling pathway. The downstream target proteins that are regulated by the active p38-MAPK signaling pathway are also regulating additional biological processes such as cell proliferation, differentiation, and apoptosis. p38-MAPK family proteins play complex and diverse roles in tumors (9, 10). In order to exert an anti-tumor effect, p38-MAPK activation may induce apoptosis and cell cycle arrest in a tumor cell (11). In addition, it has been shown that the p38-MAPK protein can regulate activity of the extracellular signal-regulated kinases 1 and 2 (ERK1/2) and the phosphoinositide 3-kinase/Akt (PI3K/AKT) signaling pathways, which in turn promotes tumor cell proliferation and growth (12). The p38-MAPK signaling pathway may promote tumor growth by regulating inflammatory responses and angiogenesis in the tumor microenvironment (11). Additionally, this pathway contributes to immunomodulation in the tumor microenvironment. The p38-MAPK signaling pathway can support immunological functions like tumor immune surveillance and immune antigen presentation by modulating the activity of immune cells and the production of immune components (13). p38-MAPK signaling pathway activation can promote the production and secretion of cytokines, such as interferon gamma (IFN-γ), interleukin 6 (IL-6), tumor necrosis factor alpha (TNF-α), and interleukin 1 (IL-1), which can promote the activation of immune cells and an immune response (6). In addition, the p38-MAPK signaling pathway can also regulate the activity of antigen-presenting cells, such as macrophages, to enhance the immune response (6). Therefore, a thorough investigation of the function of the MAP2K3 gene, which is closely associated with the p38-MAPK signaling pathway, in the immune microenvironment of glioma tumors may be helpful in understanding the mechanisms underlying glioma development and growth, and result in the development of novel therapeutic methods and targets.

In our study, MAP2K3 expression was discovered to be aberrantly high in a range of tumor tissues, and such high expression was found to be associated with poor clinicopathological characteristics and outcome of gliomas. We found that the genes related to MAP2K3 were primarily enriched in immunomodulatory pathways through functional and pathway enrichment analysis. Finally, we discovered a relationship between MAP2K3 expression and immunological checkpoints, immune-related genes, and immune infiltration in glioma. Taken together, our research highlights the critical function of MAP2K3 in tumor immune modulation and glioma prognosis; indicating the MAP2K3 gene as a potential novel target for the treatment of glioma (see Table 1).

**Table 1:** Summary of the relevant databases.

Name of database	Link
CGGA	http://www.cgga.org.cn/
CancerSEA	http://biocc.hrbmu.edu.cn/CancerSEA/
TIMRE	https://cistrome.shinyapps.io/timer/
GEPIA website	http://gepia.cancer-pku.cn/index.html
Ivy Glioblastoma Atlas Project	https://glioblastoma.alleninstitute.org/
UCSC	https://xenabrowser.net/datapage/
TIGER	http://tiger.canceromics.org/
The Human Protein Atlas	https://www.proteinatlas.org
TCGA	https://cancergenome.nih.gov/
TISCH (14)	http://tisch.comp-genomics.org/home/

## Materials and methods

### Collection of data and analysis of MAP2K3 expression

In this study, clinically relevant mRNA expression profile datasets were obtained from the public databases the Gene Expression Omnibus (GEO), the Chinese Glioma Genome Atlas (CGGA), and The Cancer Genome Atlas (TCGA). We utilized the R software for the initial processing of gene expression profiles, which encompassed tasks such as background correction, normalization, and Log 2 transformation. Subsequently, to evaluate the presence of MAP2K3 in gliomas and various other tumors, we used the TIMRE database. This allowed us to investigate MAP2K3 expression levels in tumor samples, juxtaposed with their corresponding healthy tissues. Using the GEPIA (Gene Expression Profiling Interactive Analysis) website, we also investigated the expression of MAP2K3 in low grade glioma (LGG), GBM, and normal tissues. The expression of MAP2K3 in gliomas of different WHO classifications was examined. Using the Human Protein Atlas website, we evaluated the amounts of MAP2K3 protein expression in glioma and normal brain tissues as well as the location of MAP2K3 protein in glioma cells.

### Analysis of MAP2K3 protein expression levels in gliomas

We used immunohistochemistry to evaluate the differential expression levels of MAP2K3 protein in glioma tissue compared to normal brain tissue. Images from immunohistochemical staining of normal brain tissue, LGG, and HGG were separately sourced from The Human Protein Atlas database, specifically the cerebral cortex section. The antibody used for immunohistochemistry was anti-MAP2K3 primary antibody (HPA043783).

### Evaluation of MAP2K3’s prognostic significance in glioma

In this study, both univariate and multivariate Cox regression analyses were performed to determine whether MAP2K3 might be employed as an independent prognostic factor for glioma. WHO categorization, IDH status, gender, 1p/19q code, and age were clinical variables included in the Cox regression analysis (15). The R package “rms” was used to generate column line plots and calibration. To predict overall survival (OS) at 1, 3, and 5 years, we used the “survivor” package. We utilized the “pROC” R tool to generate AUC curves for the ROC study. We additionally investigated the relationship between MAP2K3 and Overall Survival (OS) and Progress Free Survival (PFS) in several clinical cohorts with LGG and GBM.

### Gene set variation analysis

In Gene Set Variation Analysis (GSVA), the distribution of genes in a predefined set of genes is used to assess their trends in a table of phenotypically related ordered genes to determine their role in phenotype definition. To investigate the biological significance of MAP2K3, the “GSVA” package in R was used in this study to perform GSVA analysis. Based on their mRNA expression levels, MAP2K3 was split into low and high expression groups to identify the functional and pathway significance differences between the two groups. We downloaded “h.all.v7.2.symbols” and “c2.cp.kegg.v7.2.symbols” from the MsigDB database as reference gene sets for GMT (Hallmarks) and KEGG pathways, respectively. The “Limma” program was used to analyze the differences in GSVA pathways between patients in the high and low MAP2K3 groups, with adjusted criteria of *p* < 0.05 and abs (log2FC) > 0.3. Through heat maps, we displayed the Hallmarks and KEGG differential pathways individually.

### Evaluation of immunological microenvironment and tumor immune cell infiltration

In order to figure out immune, stromal, and ESTIMATE scores; our study first examined immune and stromal cell types according to gene expression profiling using the “ESTIMATE” R package. Then, we assessed the correlation between the MAP2K3 gene and various immune cell levels using the CIBERSORT and ssGSEA algorithms, and discovered a link between the level of MAP2K3 expression and the infiltration of various immune cell types.

### Evaluation of immunotherapy-related predictors

In this study, we compared the expression of several immunological checkpoints in the groups with high and low levels of MAP2K3 expression. The Wilcoxon rank sum test was used to evaluate the differences in immune checkpoint expression between the high MAP2K3-expressing and low MAP2K3-expressing groups. In order to demonstrate the sensitivity of the relevant subgroups to immune checkpoint inhibitor (ICI) therapy, we calculated the Tumor Immune Dysfunction and Exclusion TIDE (TIDE) scores for the high MAP2K3 expression group and the low MAP2K3 expression group. TIDE scores are used to assess the effectiveness of immunotherapy, with high TIDE scores indicating high tumor tolerance to immune checkpoint inhibitor therapy and low TIDE scores indicating better treatment outcomes. We then calculated the interferon gamma (IFNG) score, T cell receptor abundance (TCR), TCR Shannon score, microsatellite instability (MSI) and single nucleotide variant (SNV) neoantigens from TCGA. These metrics can be used to predict the ability of T cells in the immune microenvironment to exert tumor suppression and calculate levels of tumor neoantigens.

### Somatic cell mutation analysis

In this study, somatic mutations and copy number alterations (CNAs) were downloaded from the TCGA database, and VarScan2 software was used to whole-genome sequence data of somatic mutations in the high MAP2K3 expression group and low MAP2K3 expression group. The Fisher’s exact test was used to discover various mutation patterns, the CoMEt algorithm was utilized to find both co-occurring and mutated genes, and *p* < 0.05 was established as the threshold for choosing differentially mutated genes. For the purpose of visualizing somatic mutations, “maftools” was a R package.

### Single-cell sequencing to assess MAP2K3 expression levels in gliomas

CancerSEA and TISCH, two single-cell sequencing data platforms, were employed to evaluate MAP2K3 expression at the single-cell level in gliomas.

### *In vitro* validation of MAP2K3’s function in glioma

U251 glioma cells were cultured in Dulbecco’s Modified Eagle Medium supplemented with 10% fetal bovine serum and 1% penicillin-streptomycin, maintained at 37°C with 5% CO_2_. For gene silencing, cells underwent transfection with siRNA targeting MAP2K3 and a control siRNA from GenePharma (Shanghai, China), using Lipofectamine 3,000 as per manufacturer’s guidelines. MAP2K3 knockdown was verified by qRT-PCR 48 h post-transfection using specific primers (16). The primers used were as follows: for MAP2K3; Forward: GACTCCCGGACCTTCATCAC, Reverse: GGCCCAGTTCTGAGATGGT, and for GAPDH; Forward: TGTGGGCATCAATGGATTTGG, Reverse: ACACCATGTATTCCGGGTCAAT. The CCK-8 kit was deployed to ascertain the viability of U251 cells, as well as the survival of U251 cells.

### Wound healing assay

Cellular migration was assessed using a wound healing assay. Cells seeded in 6-well plates were grown to confluence, and a sterile 200 μL pipette tip was used to scratch a line through the monolayer. After washing away debris with phosphate-buffered saline, the cells were incubated in serum-free medium. Images were captured at 0 and 24 h post-scratch using an inverted microscope, and the rate of migration was quantified by measuring the gap with ImageJ software.

### Transwell migration assay

For the Transwell migration assay, cells were suspended in serum-free medium (1 × 10^5^ cells/mL) and 100 μL was placed into the upper chamber of a Transwell insert (Corning, United States). The lower chamber contained 600 μL of medium with 10% fetal bovine serum as a chemoattractant. Following a 24 h incubation at 37°C and 5% CO_2_, cells on the upper membrane were removed, while those on the lower surface were fixed, stained with crystal violet, and counted in five fields under a light microscope.

### Statistical analysis

The Wilcoxon test was used in this study to compare the differences in MAP2K3 expression between normal tissues and gliomas in the dataset obtained from the GEO database. We compared the variations in MAP2K3 expression in gliomas of various WHO classifications using the data acquired from TCGA and the Kruskal–Wallis test. To examine the association between survival and MAP2K3 expression levels, Kaplan–Meier curves were used.

## Results

### MAP2K3 is differentially expressed in gliomas and multiple other tumors

**Figure 1:**
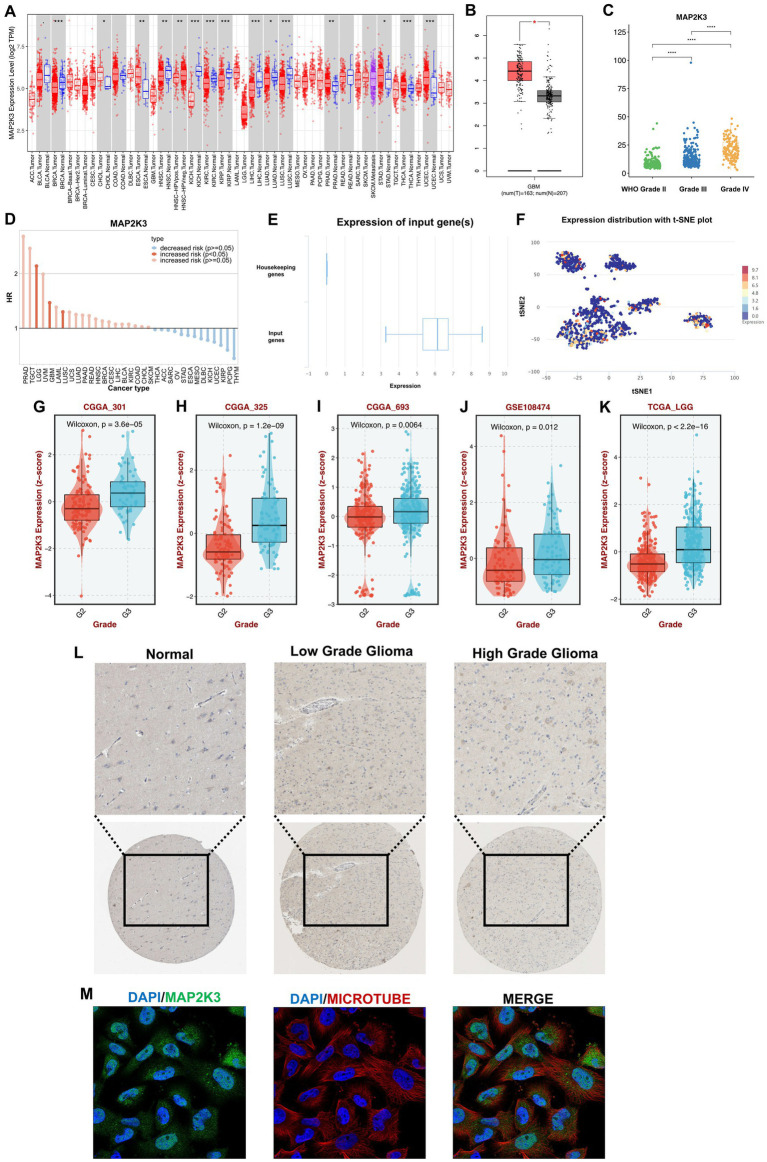
MAP2K3 is differentially expressed in gliomas. **(A)** MAP2K3 is differentially expressed in pan-cancer (*p < 0.05, **p < 0.01, and ***p < 0.001, Wilcoxon test). **(B)** MAP2K3 is differentially expressed between GBM and normal patients (*p < 0.05, **p < 0.01, and ***p < 0.001, Wilcoxon test). **(C)** MAP2K3 is differentially expressed among three WHO grades (*p < 0.05, **p < 0.01, and ***p < 0.001, Wilcoxon test). **(D)** Prognostic significance of MAP2K3 in pan-cancer. **(E)** MAP2K3 is highly expressed compared with housekeeping genes. The box plot illustrates the distribution of MAP2K3 gene expression in the glioma EXP0059 dataset. **(F)** The t-SNE plot showed the expression distribution of MAP2K3. T-SNE describes the distribution of cells, every point represents a single cell, and the color of the point represents the expression level of MAP2K3 in the cell. **(G–K)** MAP2K3 is differentially expressed in CGGA301 **(G)**, CGGA325 **(H)**, CGGA693 **(I)**, GSE108474 **(J)**, and TCGA-LGG **(K)** cohorts. **(L)** Representative images of MAP2K3 among different grades in immunohistochemistry. **(M)** Immunofluorescence staining of MAP2K3 in the SH-SY5Y cell line.

To explore the expression pattern of MAP2K3 in gliomas, we first analyzed the expression of MAP2K3 in tumor tissues. We used the TIMER database to explore the expression of MAP2K3 in 33 human cancers and found that MAP2K3 was expressed in multiple tumors (Figure 1A). The HR values of the MAP2K3 gene in LGG and GBM were higher than 1, suggesting that high MAP2K3 expression is associated with an increased risk of LGG and GBM occurrence (Figure 1D). We also used the GEPIA2 website to examine the TCGA database in order to investigate the expression of MAP2K3 in gliomas and healthy brain tissues. The findings demonstrated that GBM had higher levels of MAP2K3 mRNA expression than normal brain (Figure 1B). In addition, the expression level of MAP2K3 in gliomas correlated with the WHO grade of glioma, and the expression level of MAP2K3 in gliomas increased with the grade of glioma (Figure 1C). We found higher levels of MAP2K3 expression in multiple glioma cohorts with WHO grade 3 gliomas than WHO grade 2 (Figures 1G–K). MAP2K3 expression levels were also upregulated in the single-cell EXP0059 glioma cell group (Figures 1E,F).

We then assessed the protein expression level of MAP2K3 in gliomas using The Human Protein Atlas database. According to the results of immunohistochemical staining, glioma tissues generally express more MAP2K3 than healthy brain tissues do, and high-grade gliomas express more of the MAP2K3 protein (Figure 1L). We evaluated the localization of MAP2K3 protein in the glioma cell line SH-SY5Y, and the results showed that MAP2K3 was localized in the cytoplasm (Figure 1M). These results show that MAP2K3 is substantially expressed in both high-grade and low-grade gliomas, and that its expression level rises with increasing WHO grades. This suggests a potential association between MAP2K3 and the malignant behavior of gliomas.

### Patient prognosis is correlated with MAP2K3 expression in gliomas

**Figure 2:**
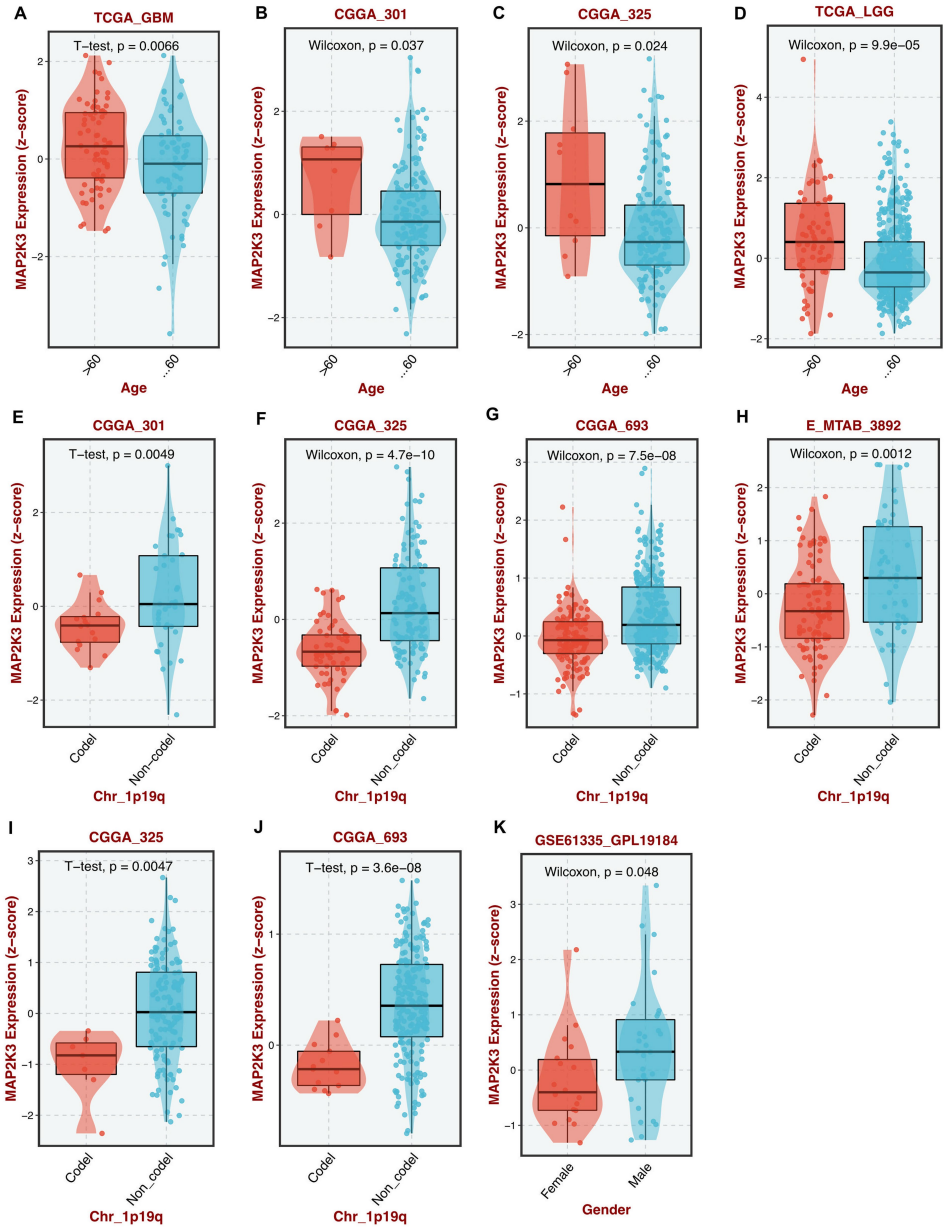
Differential expression of MAP2K3 in different clinical features. **(A–D)** MAP2K3 is differentially expressed between different ages in TCGA-GBM **(A)**, CGGA301 **(B)**, CGGA325 **(C)**, and TCGA-LGG **(D)** cohorts. **(E–J)** MAP2K3 is differentially expressed between different 1p19q status in CGGA301 **(E)**, CGGA325 **(F)**, CGGA693 **(G)**, E-MTAB-3892 **(H)**, CGGA325 **(I)**, and CGGA693 **(J)** cohorts. **(K)** MAP2K3 is differentially expressed between different genders in GSE61335 cohort (*p < 0.05, **p < 0.01, and ***p < 0.001, Wilcoxon test).

By analyzing multiple GBM cohorts and LGG cohorts, we found that MAP2K3 expression levels differed among glioma patients by age, 1p/19q co-deletion and gender; with higher MAP2K3 expression levels in young and middle-aged (<60 years), 1p/19q non-co-del, and male glioma patients (Figures 2A–K).

**Figure 3:**
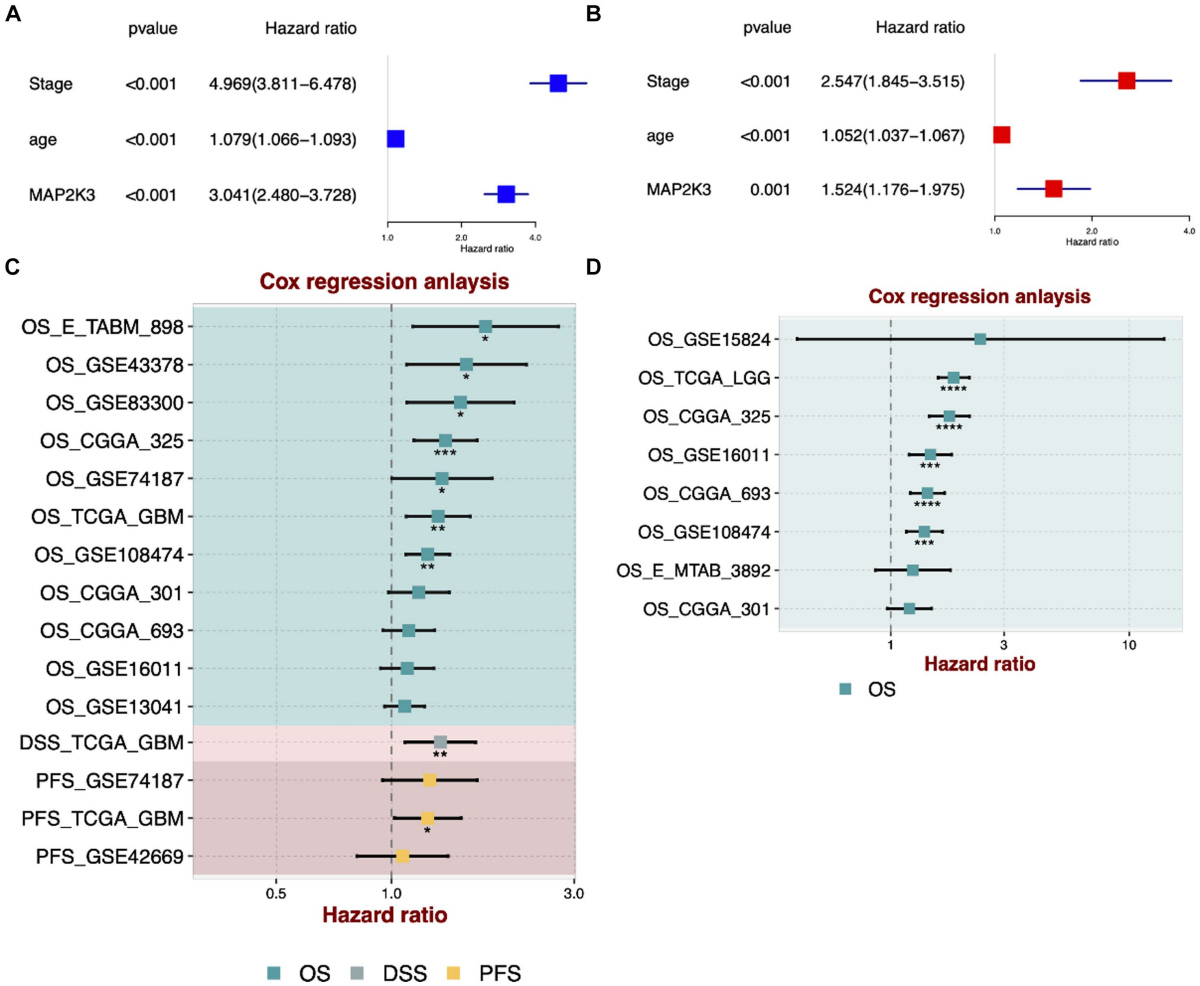
Prognostic significance of MAP2K3 in multi-center cohorts. **(A)** Univariate Cox regression analysis of Stage, age, and MAP2K3 expression. **(B)** Multivariate Cox regression analysis of Stage, age, and MAP2K3 expression. **(C)** Univariate Cox regression analysis of MAP2K3 expression in multi-center GBM cohorts. **(D)** Univariate Cox regression analysis of MAP2K3 expression in multi-center LGG cohorts.

To investigate whether high expression of MAP2K3 could be an independent predictor of glioma prognosis, univariate and multivariate Cox regression analyses were conducted. Univariate Cox regression analysis showed that MAP2K3 expression, WHO staging, and age were associated with the prognosis of glioma (Figure 3A). Multivariate Cox regression analysis revealed that MAP2K3 expression, WHO staging, and age were independent prognostic factors affecting glioma prognosis (Figure 3B). Furthermore, by performing Cox regression analysis on multiple GBM cohorts and multiple LGG cohorts, we found that the MAP2K3 gene was a significant risk factor for poor patient prognosis (Figures 3C,D).

**Figure 4:**
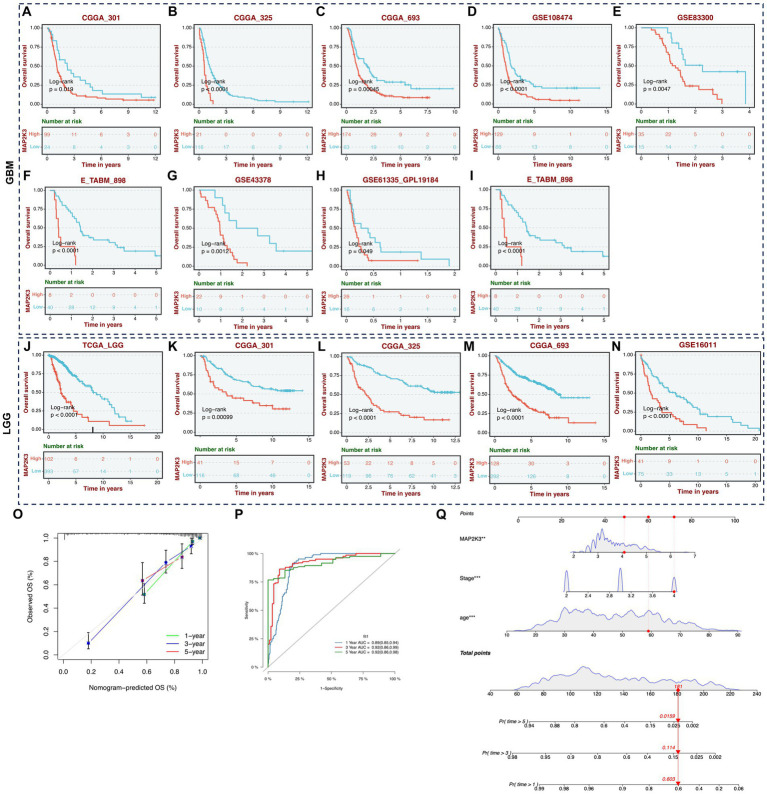
Survival analysis of expression level of MAP2K3. **(A–I)** OS Kaplan–Meier survival curves between glioma patients with high and low expression level of MAP2K3 in multi-center GBM cohorts. **(J–N)** OS Kaplan–Meier survival curves between glioma patients with high and low expression level of MAP2K3 in multi-center LGG cohorts. **(O)** Plots depicted the calibration of the nomogram. **(P)** Predictive accuracy at 1, 3, and 5 years of the nomogram in TCGA cohort. **(Q)** The nomogram plot revealed the prognostic prediction model based on MAP2K3, stage, and age in TCGA cohort.

We discovered through a multiple cohort survival study that patients with high expression levels of MAP2K3 had shorter overall survival, regardless of whether they had high- (Figures 4A–I) or low-grade gliomas (Figures 4J–N). The Nomogram and calibration curves demonstrate that MAP2K3 is an independent prognostic factor that accurately predict patient prognosis at 1, 3, and 5 years; indicating that MAP2K3 is a good predictor of prognosis for glioma patients in the multiple regression model (Figure 4O). Time-dependent analysis of ROC showed AUC values of 0.89, 0.92, and 0.92 for glioma at 1, 3, and 5 years, respectively, indicating a high predictive power (Figure 4P). As a result, MAP2K3 may be employed as a glioma diagnostic marker. These results all point to MAP2K3’s prognostic potential in gliomas.

### The potential biological mechanism of MAP2K3 in glioma

**Figure 5:**
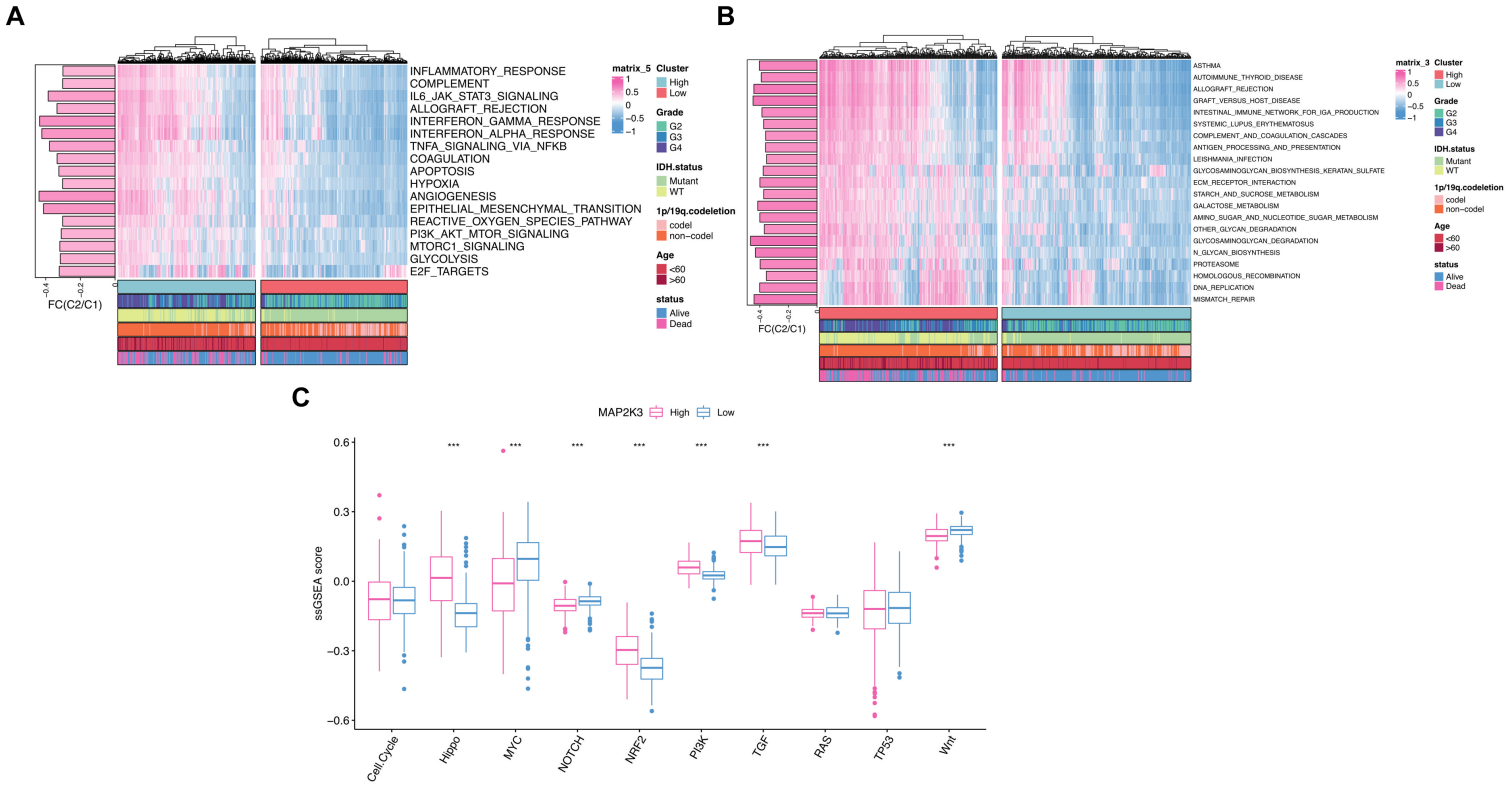
Investigations of MAP2K3-related signal pathways. **(A,B)** GSVA enrichment analyses between low and high MAP2K3 expression group illustrated the activation status of Hallmark **(A)** and KEGG **(B)** pathways in TCGA cohort. Pink and blue represent activation and inhibition of the pathway, respectively. **(C)** The Wilcoxon rank-sum test revealed the variances in the normalized scores of ten cancer-related signaling pathways between the low and high MAP2K3 expression group (*p < 0.05, **p < 0.01, and ***p < 0.001).

To investigate the potential biological mechanisms of MAP2K3 in gliomas, we explored the function of MAP2K3 molecules in multiple cancer-related signaling pathways in the TCGA cohort. To analyze Hallmarker pathway differences between gliomas with two different MAP2K3 expression levels (17), we performed GSVA gene enrichment analysis. “Inflammatory response,” “interferon gamma response,” “NF-κB/TNFA signaling pathway,” “complement,” “IL6/JAK/STAT3 signaling pathway,” and “interferon α response,” which are vital for inflammatory and immunological responses, were significantly elevated. This suggests that high MAP2K3 expression levels are closely related to immune-related signaling pathways (Figure 5A). To further explore the biological pathways of gliomas at both MAP2K3 expression levels, we performed KEGG enrichment analysis on the high MAP2K3-expressing and low MAP2K3-expressing groups of the TCGA cohort. We discovered that the group with high MAP2K3 expression was primarily related to “autoimmune thyroid disease,” “IgA-producing intestinal immune network,” “systemic lupus erythematosus,” and “antigen processing and presentation.” The MAP2K3 expression group was mainly associated with “autoimmune thyroid disease,” “IgA producing intestinal immune network,” “systemic lupus erythematosus,” “antigen processing and presentation” and other processes related to immune response (Figure 5B). These findings all point to MAP2K3’s potential involvement in immune-related pathways in gliomas.

Based on previous publications, we performed ssGSEA enrichment scoring of 10 classical oncogenic signaling pathways for two MAP2K3 expression levels in TCGA_GBM and LGG cohorts. Scoring signaling pathways including Wnt, TP53, TGF, RAS, PI3K, NRF2, NOTCH, MYC, cell cycle, and Hippo pathways. Based on the enrichment analysis outcomes, groups with higher MAP2K3 expression demonstrated elevated scores in several signaling pathways; namely the Hippo, NRF2, PI3K, and TGF pathways (Figures 5C,D). Each of these pathways are known to be closely intertwined with tumor immune evasion responses (18–22).

### MAP2K3-associated somatic mutations in glioma

**Figure 6:**
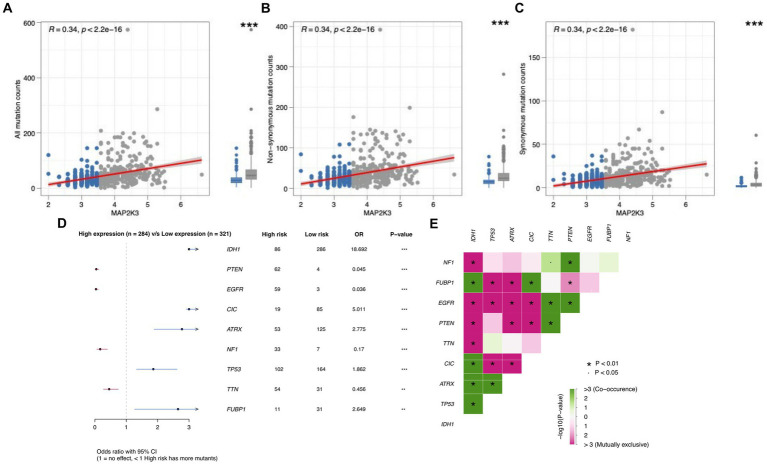
The correlation between MAP2K3 expression and tumor mutation status. **(A–C)** The relationship between all mutation **(A)**, non-synonymous **(B)**, and synonymous **(C)** counts and the MAP2K3 expression level, respectively. **(D)** Forest maps show differences of glioma patients in gene mutations in the high and low MAP2K3 expression groups. **(E)** Interaction of differentially mutated genes in glioma patients, including cooccurrence and mutual exclusion.

We analyzed somatic mutations in glioma patients from the TCGA cohort to investigate the mechanisms associated with MAP2K3 expression levels. Non-synonymous mutations are mutations that result in altered amino acid sequences (23). Some nonsynonymous mutations lead to mutations in tumor-associated genes, which may result in enhanced cell proliferation and invasiveness. Synonymous mutations are mutations in which genomic variants do not lead to amino acid sequence alterations (24). Although synonymous mutations do not directly alter the structure and function of proteins, they may affect the expression level and regulation of proteins. These aberrantly expressed proteins or peptides can be recognized by the immune system as allosteric antigens, triggering an immune response. Many tumor somatic mutations can be targets for immunotherapy and thus improve the therapeutic effect. Studying mutations in tumor cells and uncovering mutation-related molecular mechanisms can provide important references and guidance for immunotherapy and prognosis of tumors (25). In comparison to the low MAP2K3 expression group, more mutations were discovered in the high MAP2K3 expression group (Figure 6A), including non-synonymous mutations (Figure 6B) and synonymous mutations (Figure 6C).

We further analyzed the mutation frequencies of the nine most mutated genes (NF1, FUBP1, EGFR, PTEN, TTN, CIC, ATRX, TP53, IDH1) in glioma somatic cells both in the groups with high MAP2K3 expression and those with low MAP2K3 expression. Forest plots showed that IDH1, CIC, ATRX, FUBP1, and TP53 mutation frequencies were significantly higher in the low MAP2K3 expression group; while PTEN, EGFR, NF1, and TTN mutation frequencies were higher in the high MAP2K3 expression group (Figure 6D). Among them, EGFR mutations can inhibit tumor immune response through various mechanisms, such as reducing the number and function of antigen-presenting cells, decreasing T-cell infiltration and activation, and increasing the number of immunosuppressive cells (26). In addition, we observed a large number of co-occurrence of tumor-associated genes in these genes; such as NF1 and TTN, PTEN; FUBP1 and IDH1, CIC; EGFR and TTN, PTEN; PTEN and TTN; CIC and IDH1; ATRX and IDH1, TP53; TP53 and IDH1, which may indicate that these genes are interdependent or synergistic and have important roles in tumorigenesis and development (Figure 6E).

### Relationship between glioma immune cell infiltration and MAP2K3 expression level

**Figure 7:**
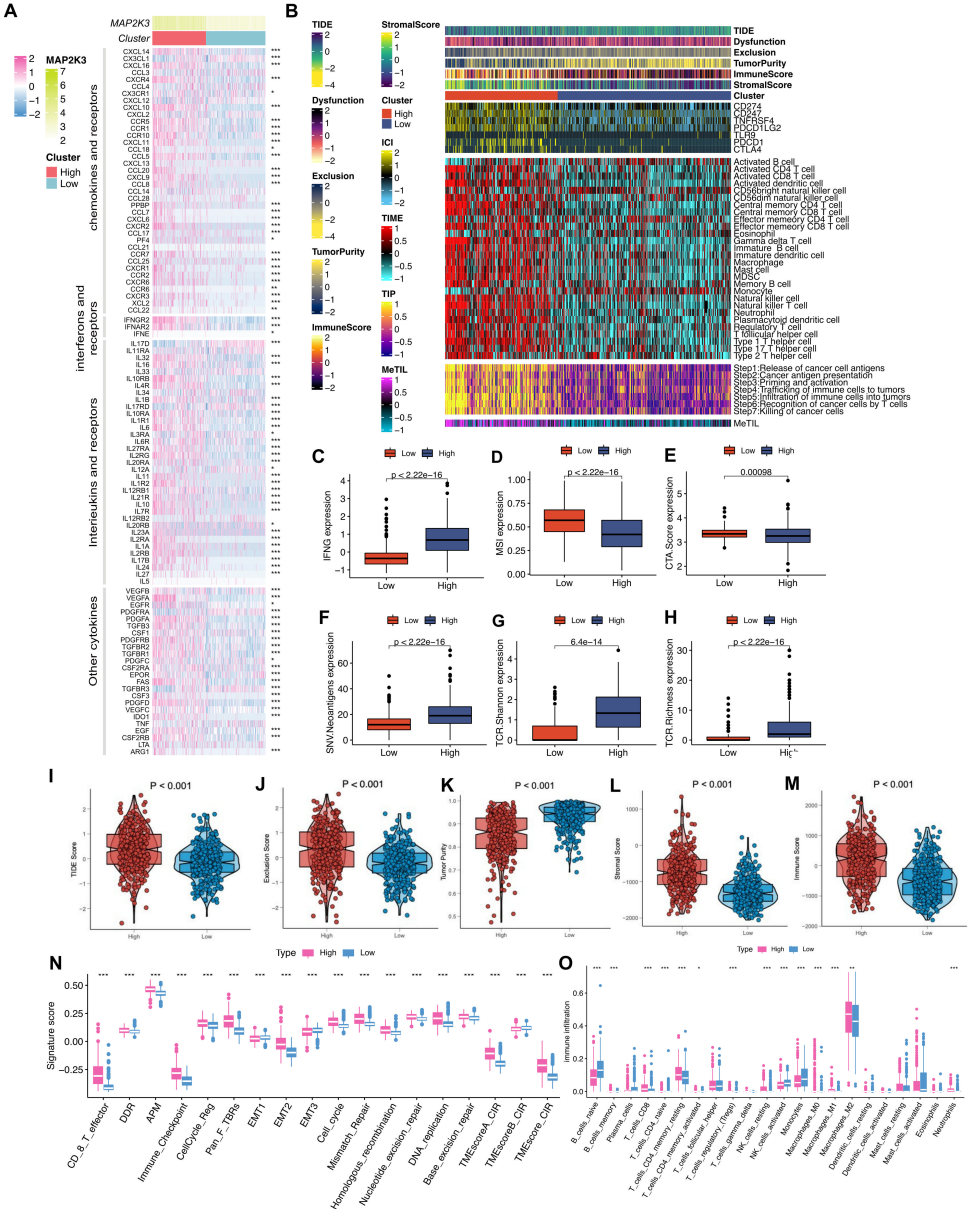
Changes in immunomodulators and quantitative types of several tumor immune microenvironments between different MAP2K3 expression groups. **(A)** Heatmap revealed changes in mRNA expression levels of chemokines and their receptors, interleukins and their receptors, interferons and their receptors, and other cytokines between two groups (*p < 0.05, **p < 0.01, and ***p < 0.001, Wilcoxon rank-sum test). **(B)** Heat maps showing the enrichment scores of immune cells between two groups in the TCGA cohort. At the left of the heatmap were annotated the TIDE score, dysfunction score, exclusion score, tumor purity, immune score, and stromal score. **(C–H)** The different level of IFN **(C)**, MSI **(D)**, CTA.Score **(E)**, SNV.Neoantigens **(F)**, TCR.Shannon **(G)**, and TCR.Richness **(H)** between the two groups. **(I–M)** The different level of TIDE score **(I)**, Exclusion score **(J)**, Tumor purity **(K)**, Stromal score **(L)**, and Immune score **(M)** between the two groups. **(N,O)** Two different MAP2K3 expression groups were distinguished by different TME-related signatures **(N)** and immune cells **(O)** (*p < 0.05, **p < 0.01, and ***p < 0.001, Wilcoxon test).

Since tumor progression and suppression are closely related to immunity, we investigated the differences in the immune microenvironment of tumors at different MAP2K3 expression levels (27, 28). First, we explored the differences in immune checkpoints and immunomodulatory factors at different MAP2K3 expression levels. By analyzing the effect of MAP2K3 expression levels on chemokines in the tumor immune microenvironment, the results showed elevated expression of several chemokines in the MAP2K3 high expression group; such as CXCL10, CCR5, CCR10, CCL5, CCL7, CCR2, and CCL22 (Figure 7A, upper part). Previous studies have shown that these chemokines exert immunosuppressive effects by attracting immunosuppressive cells, such as regulatory T cells (Tregs), macrophages, myeloid-derived suppressor cells (MDSCs) and monocytes, which may play an important role in the immune escape of gliomas. Furthermore, the analysis revealed increased expression of interferon receptors, interleukins, interleukin receptors and some other cytokines in the high MAP2K3 expression group. In contrast, immunomodulatory factor levels were significantly lower in the low MAP2K3-expressing group (Figure 7A, middle and lower part). We further calculated the overall immune cell infiltration abundance using the ssGSEA and CIBERSORT algorithms. By analyzing the gene expression patterns of immune cells, the analysis showed that more immune cells with significant immunosuppressive functions, such as Th2 cells, MDSCs, Treg cells, and M2-type macrophages, were present in the high MAP2K3 expression group, which were closely associated with immune escape, drug resistance, and poor prognosis of the tumor. Notably, CD8^+^ T cells, naive B cells, memory B cells, M1-type macrophages, resting natural killer (NK) cells and neutrophils were also enriched in the high MAP2K3-expressing group; suggesting that the group with high MAP2K3 expression has a significant number of immune cells and immunological-related factors. Therefore, the high MAP2K3-expressing group potentially could respond well to the recognition and attack of the immune system, thereby allowing for better results in immunotherapy may have better results (Figure 7B, middle part; Figure 7O).

### Relationship between the glioma’s immune microenvironment and MAP2K3 expression levels

Seven immune checkpoint molecules were expressed at higher levels in the high MAP2K3 expression group compared to the low MAP2K3 expression group, including CD274 (PD-L1), CD247, PDCD1, TNFRSF4, PDCD1LG2, TLR9, PDCD1, and CTLA4 (Figure 7B, upper part). This result suggests that the high MAP2K3 expression group expressed higher levels of immune checkpoint molecules to evade an activated anti-tumor immune response. For the antitumor immune response to effectively kill tumor cells, tumor immunotherapy must complete a series of steps and be allowed to iterate and expand, which is referred to as the tumor immune cycle. We calculated scores for the seven tumor immune cycle steps using ssGSEA. The analysis showed that all seven tumor immune cycle steps were scored higher in the high MAP2K3 expression group, which validates the important impact of the MAP2K3 gene on the tumor immune microenvironment (Figure 7B, bottom part).

We also used the TIDE algorithm and ESTIMATE algorithm to compare the relevant differences in the tumor immune microenvironment between the two MAP2K3 expression levels to quantify the association between MAP2K3 expression levels and potential immunotherapeutic effects. As can be seen, the high MAP2K3 expression group had higher stromal scores and immune scores and lower tumor purity, indicating that the high MAP2K3 expression group had a more active immune microenvironment relative to the low MAP2K3 expression group (Figure 7B, top part; Figures 7K,L). Next, using the TIDE algorithm, we found that the high MAP2K3-expressing group showed higher TIDE scores and exclusion scores (Figure 7B, top part; Figures 7I,J), which is consistent with the previous analysis, suggesting that higher TIDE scores may be related to immune escape in the high MAP2K3-expressing group.

We scored the tumor immune microenvironment by scoring patients in different MAP2K3 expression groups, and as a result, the high MAP2K3 expression group was found to have a higher exclusion score. In tumor immune microenvironment analysis, the exclusion score is typically used to evaluate whether immune cells are prevented from infiltrating the tumor tissue, thereby indirectly affecting their ability to recognize and eliminate the tumor. This higher exclusion score implies that the group with higher MAP2K3 expression would be more suitable for immunotherapy. By further analysis of the relevant indicators of immunotherapy, we found that IFN-γ, SNV neoantigens expression, the TCR Shannon score and the TCR abundance score were elevated in the high MAP2K3 expression group. All these related expression indicators were elevated suggesting that glioma patients with high MAP2K3 expression levels respond better to immunotherapy (Figures 7C–H).

In addition, we investigated biomarkers of several widely recognized immune pathways. The analysis showed that the immune-related gene sets were scored higher in the high MAP2K3 expression group, including a variety of gene sets including CD8^+^ T effector cells, DNA damage response, DNA replication, cell cycle regulation, epithelial-mesenchymal transition markers, immune checkpoints, homologous recombination, mismatch repair, and nucleotide excision repair (Figure 7N).

### Relationship between MAP2K3 expression and the TGF signaling pathway in the glioma microenvironment

**Figure 8:**
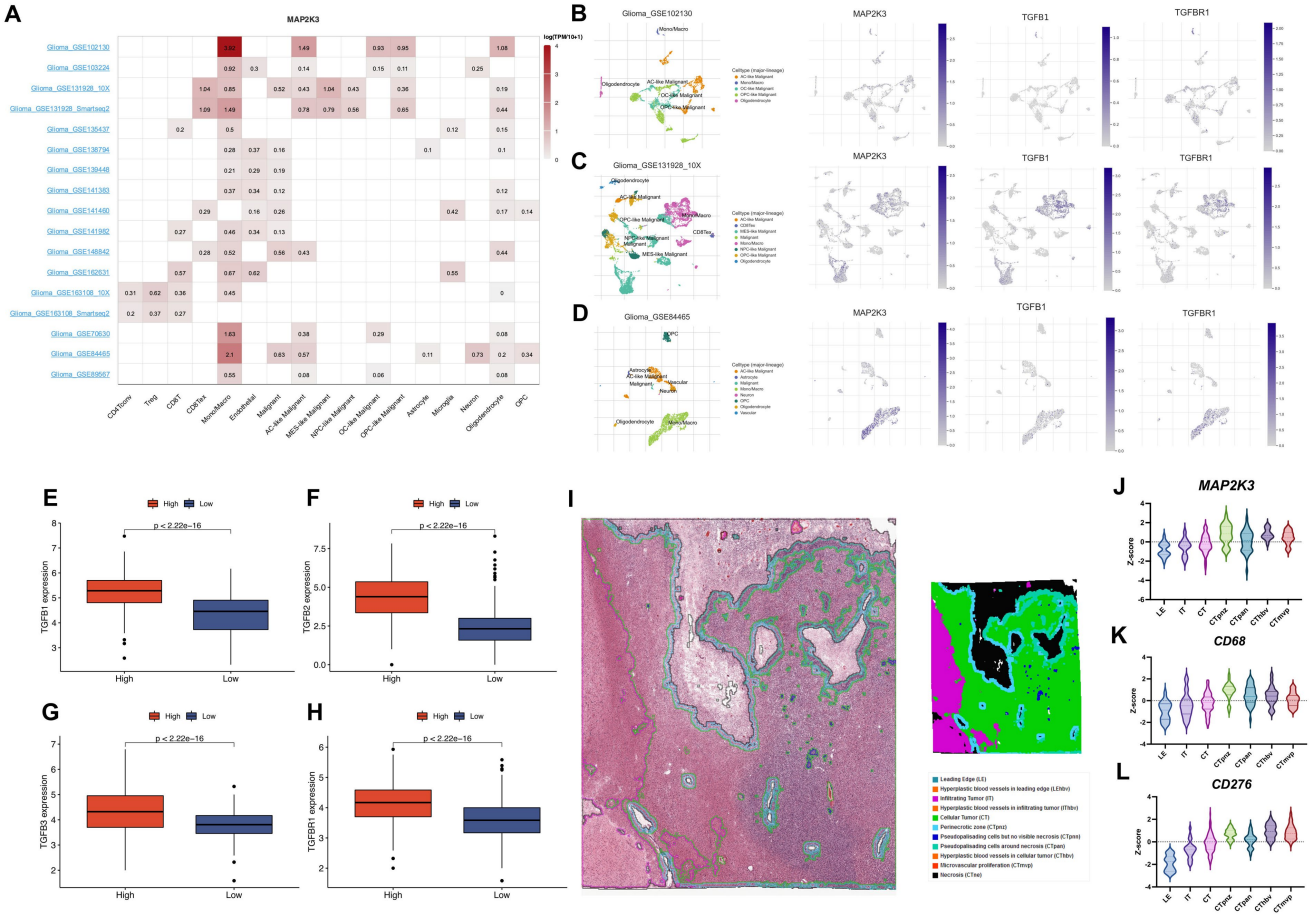
The analysis of the function and expression of MAP2K3 in single-cell RNA sequencing datasets. **(A)** The distribution of MAP2K3 expression in 17 single-cell RNA sequencing datasets. **(B–D)** The t-SNE plots showed the distribution of MAP2K3, TGFB1, and TGFBR1 expression in different cell types. **(E–H)** The expression levels of TGF beta signaling pathway between two MAP2K3 groups (Wilcoxon rank-sum test). **(I)** Annotation of tumor characteristics in human glioma samples from the Ivy Glioblastoma Atlas Project. **(J–L)** The expression level of MAP2K3, CD68, CD276 in different parts of the human glioma samples.

Previous results demonstrated that TGF-β1, TGF-β2, TGF-β3, and TGF-βR1 were expressed at higher levels in gliomas in the high MAP2K3 expression group, suggesting that high levels of MAP2K3 may convey immunosuppressive effects by activating the TGF-β signaling pathway, thereby promoting immune escape in gliomas with high MAP2K3 expression levels (Figures 8E–H).

According to the Ivy Glioblastoma Atlas Project, MAP2K3, CD68 and CD276 were found to be upregulated in human glioma samples in the Perinecrotic zone (CTpnz) region of expression. In addition, according to the Ivy Glioblastoma Atlas Project database, the distribution of MAP2K3 was consistent with the distribution of macrophage markers CD68 and CD276 (Figures 8I–L). The above analysis further demonstrates that MAP2K3 regulates the immune microenvironment through multiple signaling pathways and promotes immune escape in glioma patients, indicating the potential of MAP2K3 molecules as immunotherapeutic targets.

### Validation of MAP2K3 function and expression at the single cell RNA sequencing level

We used the single-cell RNA sequencing database TISCH to further validate MAP2K3 expression in gliomas. The analysis of 17 distinct transcriptome sequencing datasets of glioma single cells from various cell types revealed the presence of MAP2K3 (Figure 8A). MAP2K3 is expressed in various glioma cell lineages and its expression is most abundant in immune and malignant cells, of which we show representative single cell sequencing datasets (GSE102130, GSE163108_10X, and GSE148842) (Figures 8B–D). In the GSE102130 dataset, MAP2K3 was mainly expressed higher in monocytes/macrophages, astrocyte-like malignant cells (AC-like Malignant). In the GSE163108_10X dataset, MAP2K3 was mainly expressed higher in monocytes/macrophages, exhausted CD8^+^ T Cells (CD8Tex), and mesenchymal-like malignant cells (MES-like Malignant). In the GSE84465 dataset, MAP2K3 was positive in monocytes/macrophages and was positively expressed in monocytes/macrophages and neuronal cells. In addition, the distribution of MAP2K3, TGF-β1, and TGF-βR1 were concentrated in monocytes/macrophages according to the single cell sequencing analysis, which was consistent with our previous analysis. The outcomes of the single cell transcriptome sequencing study completely corroborated our earlier findings and present a macroscopic view of MAP2K3 in glioma.

### MAP2K3 facilitates the migration and invasion of glioma cells

**Figure 9:**
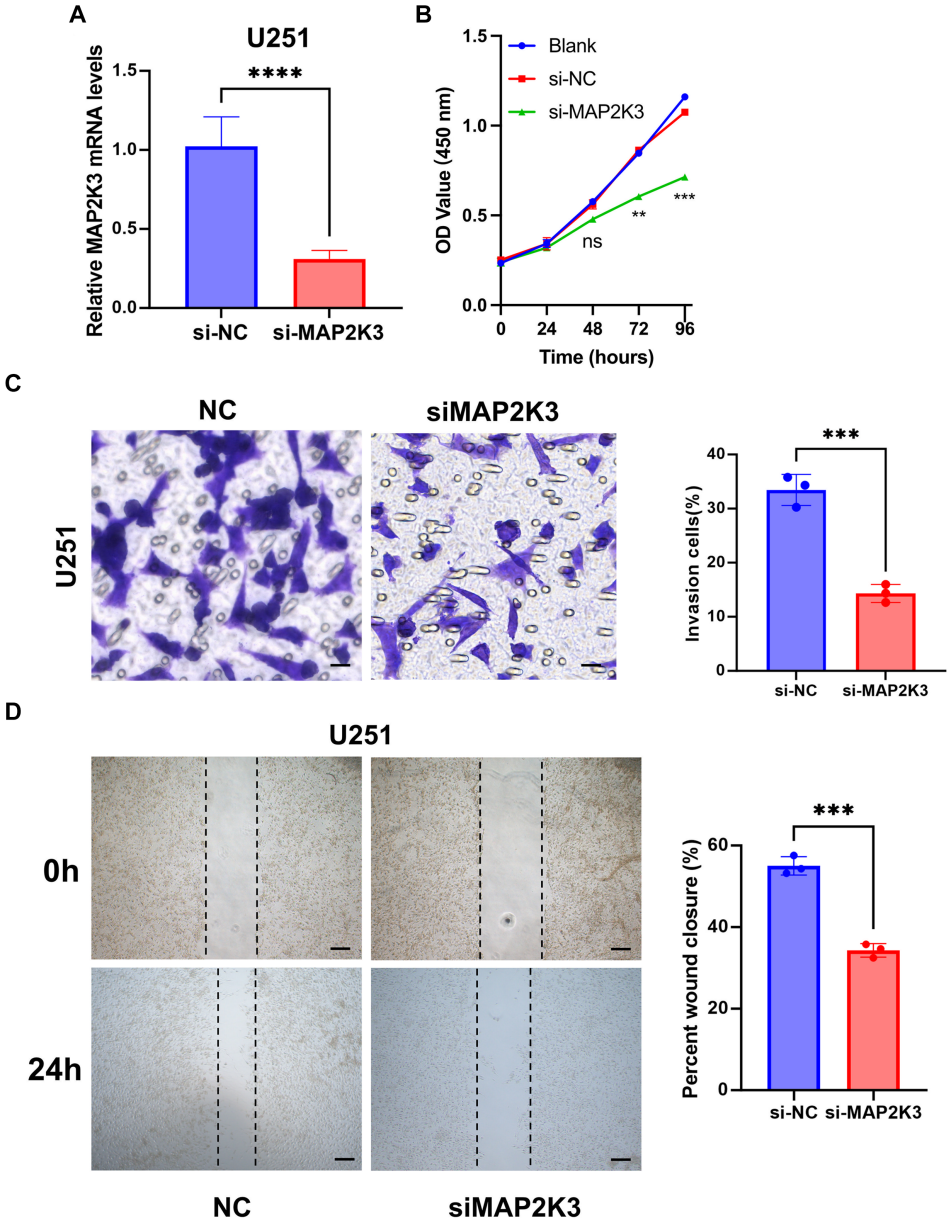
Low expression of MAP2K3 inhibited the proliferation and migration of glioma cell *in vitro*. **(A)** The mRNA expression level of MAP2K3 was effectively suppressed in U251 cells by siRNA-MAP2K3 (^****^p < 0.0001, dots represent different sample). **(B)** CCK8 assay detected cell viability after decreased MAP2K3 expression (*n* = 3). **(C)** Transwell assay detected the invasive ability of U251 cells after decreased MAP2K3 expression (*n* = 3, scale bar = 20 μM, ^***^p < 0.001). **(D)** Cell scratch assay detected the proliferation of U251 cells after decreased MAP2K3 expression (*n* = 3, scale bar = 400 μM).

We then proceeded with *in vitro* experiments to delve into the role of MAP2K3 in glioma cells. The mRNA expression of MAP2K3 was effectively suppressed in U251 cells through the use of siRNA-MAP2K3 (Figure 9A). As a result, siRNA-MAP2K3 curbed the viability of the glioma cells (Figure 9B). Analysis through wound healing demonstrated that the application of siRNA-MAP2K3 lessened the rate of wound closure compared to the siRNA control group (Figure 9D). The Transwell assay demonstrated that the invasive capacity of U251 glioma cells was reduced by MAP2K3 knockdown (Figure 9C). Taken together, these results provide strong evidence that silencing MAP2K3 notably impedes both migration and invasion of U251 cells.

### Evaluation of MAP2K3 in the effectiveness of tumor immunotherapy

**Figure 10:**
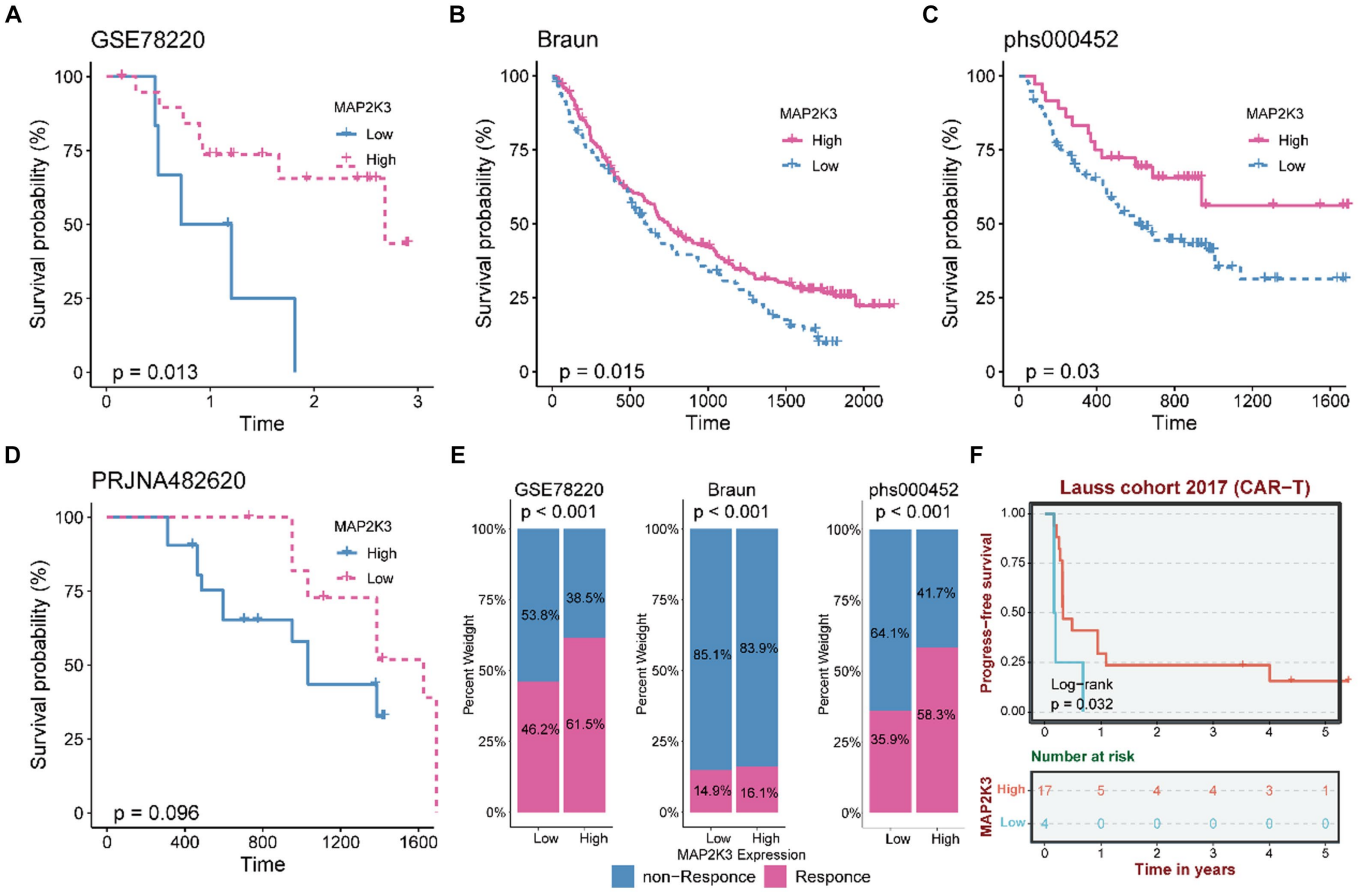
MAP2K3 predicts the response of gliomas to immunotherapy. **(A–C)** The Kaplan–Meier curve showed a significant difference in survival rate between the high and low MAP2K3 expression groups in the GSE78220 **(A)**, Braun **(B)**, and phs000452 **(C)** cohorts. **(D)** The Kaplan–Meier curve shows the relationship between survival rate and MAP2K3 expression level in PRJNA482620 cohort. **(E)** The stacked histogram shows the difference in immunotherapy responsiveness between the high and low MAP2K3 expression groups in GSE78220, Braun, and phs000452. **(F)** The Kaplan–Meier curve showed a significant difference in survival rate between the high and low MAP2K3 expression groups in the Lauss cohort.

Based on the previous assessment of MAP2K3 in the glioma tumor microenvironment, we evaluated the impact of MAP2K3 expression levels on tumor immunotherapy. In the GSE78220, Braun, and phs000452 cohorts with treatment by PD-1 inhibitors, survival curve analysis showed that the high MAP2K3 expression group had a significant prognostic advantage with longer prognostic survival after anti-PD-1 immunotherapy (Figures 10A–C). Notably, the same result was found in the PRJNA482620 cohort, an immunotherapy cohort of glioblastoma patients. Small sample sizes may have led to insignificant statistical analysis (Figure 10D). Simultaneously, the high MAP2K3 expression group in the GSE78220, Braun, and phs000452 cohorts indicated more effective anti-PD-L1 immunotherapy (Figure 10E). Additionally, in the Lauss 2017 cohort study exploring predictors and biomarkers in CAR-T therapy, the high MAP2K3-expressing group had a prognostic advantage (Figure 10B). The above results suggest that patients in the high MAP2K3 expression group may derive more benefit from tumor immune checkpoint inhibitor therapy.

## Discussion

Glioma is a common primary intracranial malignancy in adults, of which glioblastoma is more malignant and the most aggressive and incurable type with high recurrence rate and high mortality. Traditional treatments such as surgery, radiotherapy and chemotherapy have reached a bottleneck and therefore there is an urgent need to find new treatment methods. The advent of immunotherapy and targeted therapy offers new hope for glioma patients.

In previous studies, MAP2K3 was identified as an oncogene, and reducing MAP2K3 expression could reduce the rate of tumor growth and improve the biological response to chemotherapy. In our study, based on an individual dataset of 33 tumors in the TCGA database, we analyzed the expression of MAP2K3 in different types of cancers. Data analysis showed that MAP2K3 expression differed in a variety of tumors, and the results of applying the Cox Proportional-Hazards model suggested that high MAP2K3 expression was associated with an increased risk of LGG and GBM development. By analyzing the TCGA database, multiple clinical study cohorts and single cell sequencing sets, we found that MAP2K3 mRNA expression was elevated in gliomas and closely correlated with tumor grade. In addition, by analyzing multiple cohorts, we found that MAP2K3 expression levels in gliomas correlated with various clinical characteristics such as age, gender, and 1p/19q mutation status. Among them, MAP2K3 expression levels were higher in male patients, young and middle-aged glioma patients, and glioma patients with 1p/19q present. By performing univariate and multivariate Cox regression analyses, we found that high MAP2K3 expression was an independent predictor of prognosis in glioma patients. It was also established by analysis of survival data from numerous clinical cohorts that glioma patients with high MAP2K3 expression had considerably shorter survival periods and shorter progression-free survival than those with low expression. These findings imply that MAP2K3, which may be utilized to predict the prognosis of gliomas by MAP2K3 expression levels, is directly implicated in the biological malignancy of glioma. The mechanism by which MAP2K3 may function biologically in gliomas is not yet known.

Therefore, to investigate the potential biological mechanisms of MAP2K3 in glioma, we explored the functions of MAP2K3 molecules in various cancer-related signaling pathways in the TCGA cohort by GSVA gene enrichment analysis, which showed that several signaling pathways related to immune response and inflammation were significantly activated in gliomas with high MAP2K3 expression levels. We performed KEGG enrichment analysis on the high MAP2K3-expressing and low MAP2K3-expressing groups in the TCGA cohort and found that the high MAP2K3-expressing group was mainly associated with immune response and DNA repair pathways, and all these results suggest that high MAP2K3 expression levels are closely related to immune-related signaling pathways in gliomas. By ssGSEA enrichment scoring of 10 classical oncogenic signaling pathways, we found that the Hippo signaling pathway, NRF2 signaling pathway, PI3K signaling pathway and TGFβ signaling pathway scored higher in the high MAP2K3 expression group.

Given that the TGF signaling pathway is crucial for controlling immune cell activity and tumor immune escape, we specifically focused on the relationship between TGF-β1, TGF-β2, TGF-β3, TGF-βR1, and this MAP2K3 expression level in the TGF signaling pathway. In the tumor microenvironment, activation of the TGF signaling pathway can inhibit the function of immune cells, thus protecting tumors from the immune system (29–31). TGF-β1, TGF-β2, and TGF-β3 are three isoforms of the TGF-β family, which are the most important ligands in the TGF signaling pathway (22). In tumors, ligands of the TGF-β family often lose their normal functions, thus promoting tumor proliferation, metastasis, and invasion. TGF-βR1 is an important signaling molecule in the TGF-β signaling pathway, and it is a subtype of TGF-β receptor type I. TGF-βR1 can activate a variety of downstream signaling pathways through phosphorylation and kinase activation, thereby regulating cell proliferation, apoptosis, cell cycle and many other biological processes (32). In tumors, abnormal expression and uncontrolled function of TGF-βR1 can affect the normal function of the TGF signaling pathway, thus promoting tumor growth and development.

Current studies have shown that MAP2K3 plays an important role in regulating the TGF-β signaling pathway. In the TGF-β signaling pathway, TGF-β, upon binding to its cell membrane receptor, activates Smad proteins and allows them to enter the nucleus, thereby regulating gene transcription (33). In contrast, MAP2K3, a mitogen-activated protein kinase (MAPK) kinase activator, activates JNK and p38 MAPK, members of the MAPK family associated with the regulation of Smad proteins. TGFB1, TGFB2, TGFB3, and TGFBR1 expression in the TGFβ signaling pathway were higher in the high MAP2K3 expression group. This suggests that high levels of MAP2K3 may convey immunosuppressive effects by activating the TGFβ signaling pathway, thus promoting immune escape in gliomas with high MAP2K3 expression levels. Somatic mutations may be an important predictor of tumor response to immunotherapy. By analyzing somatic mutations in glioma patients in the TCGA cohort, we found more somatic mutations in the high MAP2K3-expressing group. These results suggest that there may be strong immune escape in the high MAP2K3-expressing group.

Another important finding of this study was the correlation between MAP2K3 expression and the level of immune infiltration in gliomas. Tumor cells tend to evade cytotoxic T lymphocytes by upregulating immune checkpoint ligands, such as PD-L1. MAP2K3 may regulate immunity in gliomas by interacting with or modulating immune checkpoints. Immunological checkpoint inhibitors can be used to suppress immunological checkpoint expression, stimulate T cells, and alter the tumor microenvironment. However, it is the high tumor heterogeneity, the altered expression of immune checkpoints and the widespread presence of a suppressive tumor immune microenvironment in tumors that make immunotherapy in glioma more challenging. Therefore, it is important to predict the likely therapeutic outcome of patients to immune checkpoint inhibitor therapy. We found elevated expression of multiple immunosuppressive chemokines, increased abundance of multiple immune cell infiltrates with immunosuppressive functions, and higher expression levels of seven immune checkpoint molecules in the high MAP2K3-expressing group; including CD274 (PD-L1), CD247, PDCD1, TNFRSF4, PDCD1LG2, and TLR9. The high expression of these immune checkpoints suggests that glioma patients have a poor prognosis due to the immune escape phenomenon. In addition, multiple tumor immune microenvironment scores were higher in the high MAP2K3 expression group, all suggesting a more active immune microenvironment in glioma patients with high levels of MAP2K3 expression and possibly a better response to immunotherapy. By analyzing multiple clinical cohorts, we evaluated the impact of MAP2K3 expression levels on tumor immunotherapy and found a significant prognostic advantage in the high MAP2K3 expression group after anti-PD-1 immunotherapy, which is consistent with our predicted results. All of these results suggest that patients in the high MAP2K3-expressing group may derive more benefit from tumor immune checkpoint inhibitor therapy.

There are several shortcomings of this study: First, MAP2K3 expression was only validated at the mRNA level, not at the protein level or cellular level, and *ex vivo* experiments are needed to further validate the possible signaling pathways involved in MAP2K3 in glioma. Second, the mechanistic studies in this study were all concomitant phenomena and did not demonstrate a causal association between the two. In addition, this study needs to further expand the sample size and increase the number of study centers to further enhance the significance of the results.

In conclusion, we found that MAP2K3 is expressed at high levels in gliomas and that high MAP2K3 expression predicts poor prognosis in glioma patients. This study also suggests that MAP2K3 may be a novel biomarker for prognosis prediction and immune checkpoint inhibitor therapy in glioma. In addition, patients with high MAP2K3 expression may be considered for immune checkpoint inhibitors treatment.

## Conclusion

In summary, we observed a marked overexpression of MAP2K3 in gliomas, correlating with the WHO classification system. Higher levels of MAP2K3 were found to be linked with poor outcomes in low-grade gliomas, gliomas across all WHO grades, as well as recurrent tumors. Additionally, MAP2K3 expression in gliomas displayed associations with a range of immune cells, and showed links with numerous immune checkpoints, implying its potential significance in determining patient responsiveness to immunotherapy. Furthermore, MAP2K3 bore associations with immune-related pathways. These insights point towards the potential of MAP2K3 as a prognostic indicator for gliomas, and predictive biomarker for immunotherapy responses.

## Data availability statement

The raw data supporting the conclusions of this article will be made available by the authors, without undue reservation.

## Ethics statement

Ethical approval was not required for the studies on humans in accordance with the local legislation and institutional requirements because only commercially available established cell lines were used.

## Author contributions

BP: Writing – review & editing, Writing – original draft. SF: Writing – review & editing, Software, Formal analysis. LG: Writing – review & editing, Supervision. DS: Writing – review & editing, Investigation. ZJ: Writing – review & editing, Conceptualization. XX: Writing – review & editing, Resources, Project administration, Funding acquisition. LW: Writing – review & editing.
